# Genomic regions associated with herbicide tolerance in a worldwide faba bean (*Vicia faba* L.) collection

**DOI:** 10.1038/s41598-021-03861-0

**Published:** 2022-01-07

**Authors:** Lynn Abou-Khater, Fouad Maalouf, Abdulqader Jighly, Alsamman M. Alsamman, Diego Rubiales, Nicolas Rispail, Jinguo Hu, Yu Ma, Rind Balech, Aladdin Hamwieh, Michael Baum, Shiv Kumar

**Affiliations:** 1 International Center for Agricultural Research in the Dry Areas (ICARDA), Terbol, Lebanon; 2 Agriculture Victoria, Victoria, Australia; 3 Molecular Genetics and Genome Mapping, Agricultural Genetic Engineering Research Institute, Giza, Egypt; 4 African Genome Center, Mohammed VI Polytechnic University, Ben Guerir, Morocco; 5 Institute for Sustainable Agriculture, CSIC, 14004 Córdoba, Spain; 6 USDA-ARS Plant Germplasm Introduction & Testing Research Unit, Pullman, USA; 7 Department of Horticulture, Washington State University, Pullman, USA; 8 ICARDA, Rabat, Morocco; 9 ICARDA, Cairo, Egypt

**Keywords:** Biotechnology, Genetics, Plant sciences

## Abstract

Weeds represent one of the major constraints for faba bean crop. The identification of molecular markers associated with key genes imparting tolerance to herbicides can facilitate and fasten the efficient and effective development of herbicide tolerant cultivars. We phenotyped 140 faba bean genotypes in three open field experiments at two locations in Lebanon and Morocco against three herbicide treatments (T1 metribuzin 250 g ai/ha; T2 imazethapyr 75 g ai/ha; T3 untreated) and one in greenhouse where T1 and T3 were applied. The same set was genotyped using genotyping by sequencing (GBS) which yield 10,794 high quality single nucleotide polymorphisms (SNPs). ADMIXTURE software was used to infer the population structure which revealed two ancestral subpopulations. To identify SNPs associated with phenological and yield related traits under herbicide treatments, Single-trait (ST) and Multi-trait (MT) Genome Wide Association Studies (GWAS) were fitted using GEMMA software, showing 10 and 14 highly significant associations, respectively. Genomic sequences containing herbicide tolerance associated SNPs were aligned against the NCBI database using BLASTX tool using default parameters to annotate candidate genes underlying the causal variants. SNPs from acidic endochitinase, LRR receptor-like serine/threonine-protein kinase RCH1, probable serine/threonine-protein kinase NAK, malate dehydrogenase, photosystem I core protein PsaA and MYB-related protein P-like were significantly associated with herbicide tolerance traits.

## Introduction

Faba bean (*Vicia faba* L.), also known as broad bean, fava bean, horse bean and field bean, was first domesticated in the Near East around 9000–10,000 BC^1–3^. The recent estimates suggest that it is extensively grown on 2.57 M ha area distributed across 38 countries with global production of 5.4 million tonnes^4^. Faba bean is an important source of food and feed for human and animal consumption because its seeds are rich in proteins, carbohydrates, fibers and micronutrients^5^. Faba bean plays an important role in sustainable agriculture and ecosystem services because of its ability to improve soil fertility by fixing atmospheric nitrogen^6^, and its potential to enhance the grain yield of succeeding/companion crops when planted in rotation or intercropped with cereals^7^. Concerted efforts have been undertaken to improve yield, adaptation to different environments, tolerance to abiotic stresses including heat, drought, waterlogging and frost^8–10^ resistance to biotic stresses such as diseases, insect pests, viruses and parasitic weeds^11,12^, seed quality^13^ and other agronomic traits. These efforts have more than doubled the global average yield from 0.9 tonnes/ha in 1964 to 2.1 tonnes/ha in 2019^4^. However, the current production remains insufficient to meet its global consumption. Faba bean performance is highly influenced by environments and genotype × environment (GE) interaction, making phenotypic selection for quantitative traits of breeders’ interest inefficient and cumbersome.

Faba bean has a relatively large genome size of 13 Gb^14^. Thanks to the advances in the next generation sequencing technologies (NGS) that has enabled the generation of large volumes of sequences^15–17^ and facilitated the discovery of single nucleotide polymorphisms (SNPs) that can be associated with key breeding traits either through biparental mapping or through genome wide association studies (GWAS)^15,18^. Unlike biparental mapping, GWAS utilizes natural populations and exploits linkage disequilibrium (LD) to detect SNP-trait associations with higher resolution^19^. However, the power of GWAS depends on the size and structure of the population used for the analysis^20^. While it is sometimes not feasible to phenotype large populations in a single field trial, multiple field trials, e.g. different treatments, locations or seasons, can be jointly analyzed in one model named as multi-variate or multi-trait GWAS which has shown to have higher power compared to the standard single-trait GWAS^21^. Such approach can assist conventional breeding by implementing marker assisted selections in early generations^18,22^. Although significant progress has been made in faba bean genomics and many genetic maps are available^23–25^, the marker density of most of them is still too low to enable accurate prediction of desired traits. SNPs correlated with traits of interest such as resistance to ascochyta and broomrape or vicine-convicine content^26–29^ have been identified, however, no study was conducted to associate SNPs with herbicide tolerance in faba bean.

Weeds are among the difficult-to-control biotic stresses that affect faba bean^30^. When weeds are left uncontrolled, they cause severe loss on grain yield of up to 70%^31^. An integrated approach with many control measures has been recommended to provide protection against weeds^32–34^ but with limited success. Many studies have acknowledged breeding for weed resistance by selecting for morphological characteristics that promote competition and allelopathy such as early seedling emergence, seedling growth, greater plant height, greater root volume^35–37^, but the resistance against most parasitic weeds is a difficult task because of its complex nature and low heritability^38,39^. Thus, recent studies have focused on developing herbicide tolerant faba bean lines^40,41^. Abou-Khater et al.^42^ evaluated faba bean germplasm for traits associated with tolerance to metribuzin and imazethapyr, two herbicides commonly available that can control the majority of weeds threatening faba bean production. They found that crop phenology, plant architecture and grain yield related traits were greatly affected by the herbicide treatments. Although useful sources for herbicide tolerance were identified by the authors, such field techniques are very laborious and require multi-environmental data. Associating the herbicide tolerance related traits^42^ with molecular markers to select for herbicide tolerance would facilitate the detection of useful markers that can be used to select herbicide tolerant lines in early generations. Keeping this in mind, the present study was undertaken to identify candidate loci significantly associated with tolerance to two post emergence herbicides, namely metribuzin and imazethapyr under different environments using GWAS and to identify associated SNP markers that can be used for introgressing such traits into desired agronomic background.

## Results

### Phenotyping

Multiple environmental models were fitted to obtain the best linear unbiased prediction (BLUP) values for each genotype and treatment across field trials. The genotypic effects for all studied traits and reduction indexes were significant across trials at a *p-*value < 0.001 except for the RI_GCC_ (Table 1) indicating a wide range of genotypic variation in faba bean. Significant differences were observed among treatments for all studied traits and reduction indexes except for RI_PLHT_, RI_GYPLT_ and RI_NPPLT_; while significant Genotype × Treatment interactions were observed across trials for DFLR, DMAT, PLHT, GYPLT and NSPLT (Table 1). The Genotype × Treatment × Environment interactions show that the effect of herbicide treatments on the traits and reduction indexes of the genotypes was not affected by the environment except for DFLR and NSPLT and their reduction indexes (Table 1). As for the greenhouse experiment, the DFLR, PLHT and GCC varied significantly among genotypes and treatments and significant Genotype × Treatment interactions were observed. The reduction indexes for DFLR, PLHT and GCC varied significantly also among genotypes (Table 2). Our results showed that both herbicide treatments affected the faba bean phenology by delaying significantly the DFLR and DMAT (Tables 1, 2). In addition, the post emergence application of metribuzin and imazethapyr affected the architecture of the faba bean plants by reducing the PLHT and the GCC and increasing the NbrPLT (Tables 1, 2). Moreover, a significant reduction in the GYPLT, NPPLT and NSPLT of the genotypes treated with metribuzin or imazethapyr was observed across trials. The plant height recorded in the green house experiment at two different stages showed that the treated plants tend to recover from the herbicide effect (Tables 1, 2).

**Table 1:** Combined analysis performed for detecting differences among faba bean genotypes (Geno), herbicide treatments (Trt), Geno × Trt interaction, Genotype × Environmnent (Geno × Env) interaction and Geno × Trt × Env interaction expressed as p-value and means ± standard error (SE) and ranges of the genotypes under trials.

		*p-*value	Metribuzin 250 g ai/ha		Imazethapyr 75 g ai/ha		Control		SE
			Mean	Range	Mean	Range	Mean	Range	
DFLR (DAS)	Geno	< 0.001	99.51	90.96–121.39	99.44	90.48–121.61	98.10	90.60–117.94	2.57
	Trt	< 0.001							
	Geno × Trt	< 0.001							
	Geno × Env	< 0.001							
	Geno × Trt × Env	< 0.001							
DFLR_RI	Geno	< 0.001	− 2.37	− 10.22–3.57	− 2.31	− 11.90 to 3.95			2.71
	Trt	0.749							
	Geno × Trt	1							
	Geno × Env	< 0.001							
	Geno × Trt × Env	< 0.001							
DMAT (DAS)	Geno	< 0.001	162.92	159.20–167.00	161.88	158.30–167.00	161.05	158.00–166.80	1.17
	Trt	< 0.001							
	Geno × Trt	< 0.001							
	Geno × Env	< 0.001							
	Geno × Trt × Env	0.036							
DMAT_RI	Geno	< 0.001	− 1.63	− 4.23 to 0.13	− 1.07	− 3.14 to 0.13			0.86
	Trt	< 0.001							
	Geno × Trt	0.22							
	Geno × Env	< 0.001							
	Geno × Trt × Env	0.970							
PLHT (cm)	Geno	< 0.001	61.61	37.28–83.24	60.73	32.40–83.09	73.44	36.29–104.09	6.56
	Trt	< 0.001							
	Geno × Trt	< 0.001							
	Geno × Env	< 0.001							
	Geno × Trt × Env	0.002							
PLHT_RI	Geno	< 0.001	13.93	− 41.62–37.27	17.18	− 1.16 to 43.90			11.36
	Trt	0.002							
	Geno × Trt	0.997							
	Geno × Env	< 0.001							
	Geno × Trt × Env	0.809							
GYPLT (g)	Geno	< 0.001	14.68	− 2.37 to 36.82	14.55	− 1.73 to 40.57	20.37	1.25–41.94	5.59
	Trt	< 0.001							
	Geno × Trt	< 0.001							
	Geno × Env	< 0.001							
	Geno × Trt × Env	0.190							
GYPLT_RI	Geno	< 0.001	15.00	− 38.59 to 58.94	20.78	− 50.26 to 65.45			25.78
	Trt	0.105							
	Geno × Trt	0.999							
	Geno × Env	0.103							
	Geno × Trt × Env	0.787							
NPPLT	Geno	< 0.001	17.41	6.56–36.72	16.99	8.65–29.18	19.43	7.62–44.13	4.76
	Trt	< 0.001							
	Geno × Trt	0.587							
	Geno × Env	< 0.001							
	Geno × Trt × Env	0.437							
NPPLT_RI	Geno	< 0.001	4.04	− 93.79 to 78.52	5.22	− 82.43 to 49.77			32.59
	Trt	0.393							
	Geno × Trt	1							
	Geno × Env	< 0.001							
	Geno × Trt × Env	1							
NSPLT	Geno	< 0.001	20.38	1.54–34.68	21.94	2.28–44.85	28.82	2.36–63.65	6.91
	Trt	< 0.001							
	Geno × Trt	< 0.001							
	Geno × Env	< 0.001							
	Geno × Trt × Env	< 0.001							
NSPLT_RI	Geno	0.016	24.38	− 33.02 to 75.63	14.1	− 53.60 to 60.39			31.82
	Trt	< 0.001							
	Geno × Trt	1							
	Geno × Env	< 0.001							
	Geno × Trt × Env	0.007							
NBrPLT	Geno	< 0.001	4.16	0.68–8.25	3.31	0.98–6.12	3.14	0.77–7.12	1.46
	Trt	< 0.001							
	Geno × Trt	0.931							
	Geno × Env	ND							
	Geno × Trt × Env	ND							
NBrPLT_RI	Geno	< 0.001	− 50.49	− 308.98 to 53.28	− 18.43	− 307.77 to 49.14			78.70
	Trt	< 0.001							
	Geno × Trt	1							
	Geno × Env	1							
	Geno × Trt × Env	ND							
GCC	Geno	< 0.001	29.35	− 0.89 to 59.23	25.18	1.71–53.46	34.47	3.12–67.22	11.93
	Trt	< 0.001							
	Geno × Trt	0.995							
	Geno × Env	ND							
	Geno × Trt × Env	ND							
GCC_RI	Geno	0.094	5.79	− 180.80 to 93.46	16.79	− 376.14 to 87.11			60.34
	Trt	0.027							
	Geno × Trt	0.954							
	Geno × Env	ND							
	Geno × Trt × Env	ND							

*DFLR* days to flowering, *DAS* days after sowing, *DFLR_RI* DFLR reduction index, *DMAT* days to maturity, *DMAT_RI DMAT* reduction index, *PLHT* plant height, cm centimeter, *PLHT_RI* reduction index of PLHT, *GYPLT* grain yield per plant,g gram, *GYPLT_RI* GYPLT reduction index, *NPPLT* number of pods per plant, *NPPLT_RI* NPPLT resuction index, *NSPLT* number of seeds per plant, *NSPLT_RI* NSPLT reduction index, *NBrPLT* number of branches per plant, *NBrPLT_RI* NBrPLT reduction index, *GCC* green canopy cover, *GCC_RI* green canopy cover reduction index, *ND* no data.

**Table 2:** Analysis of Variance performed for detecting differences among faba bean genotypes (Geno), herbicide treatments (Trt), and Geno × Trt interaction for different traits and reduction indexes, expressed as p- value and means ± standard error (SE) and ranges of the genotypes under different treatments in the pot trial.

		*p-*value	Metribuzin @250 g ai/ha		Control		SE
			Mean	Range	Mean	Range	
DFLR	Geno	< 0.001	56.65	38.93–62.21	49.04	34.94–61.80	7.12
	Trt	< 0.001					
	Geno × Trt	< 0.001					
DFLR_RI	Geno	< 0.001	2.27	− 65.37 to 49.79			18.26
GCC	Geno	< 0.001	5.09	− 0.27 to 10.47	6.95	2.37–10.74	1.78
	Trt	< 0.001					
	Geno × Trt	< 0.001					
GCC_RI	Geno	< 0.001	18.62	− 237.69 to 104.02			40.66
PLHT_1	Geno	< 0.001	15.12	2.63–26.27	25.29	11.55–42.65	4.70
	Trt	< 0.001					
	Geno × Trt	< 0.001					
PLHT_RI_1	Geno	< 0.001	28.17	− 73.02 to 89.70			19.2
PLHT_2	Geno	< 0.001	21.23	5.16–35.67	35.90	17.97–58.62	7.26
	Trt	< 0.001					
	Geno × Trt	0.004					
PLHT_RI_2	Geno	< 0.001	53.78	− 34.25 to 100.00			26.05

*DFLR* days to flowering, *DFLR_RI* DFLR reduction index, *GCC* green canopy cover, *GCC_RI* green canopy cover reduction index, *PLHT_1* plant height recorded at flowering (BBCH code 60), *PLHT_1_RI* reduction index of PLHT_1, *PLHT_2* plant height recorded at pod development (BBCH code 70), *PLHT_2_RI* PLHT_2 reduction index.

**Figure 1:**
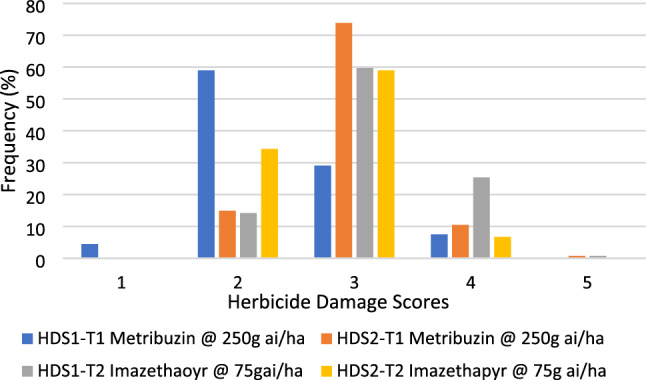
Distribution of faba bean genotypes for herbicide damage scores (HDS1 and HDS2) under metribuzin at 250 g ai/ha and imazethapyr at 75 g ai/ ha.

Our results also showed that the first (HDS1) and second (HDS2) herbicide damage scores per genotype varied from 1 to 5 across trials. Combined results of the herbicide damage scores (HDS1 and HDS2) showed that after one month of the herbicide application, 5 and 42% genotypes recovered from the damaged caused by metribuzin and imazethapyr treatments while damages in 56 and 10% genotypes exacerbated (Fig. 1). The herbicide damage on the remaining genotypes remained unchanged between the first and second recording dates.

**Figure 2:**
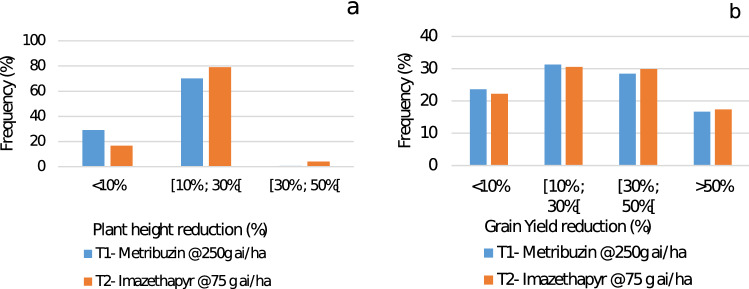
Distribution of faba bean genotypes for plant height reduction PLHT_RI (**a**) and grain yield per plant reduction GYPLT_RI (**b**) under metribuzin at 250 g ai/ha and imazethapyr at 75 g ai/ ha.

Figure 2 shows that the herbicide treatments affected differently the plant height and grain yield of the treated genotypes. The reduction in plant height and grain reduction varied between almost negligeable (< 10%) and high levels (> 40%).

### Genotyping and population structure

**Figure 3:**
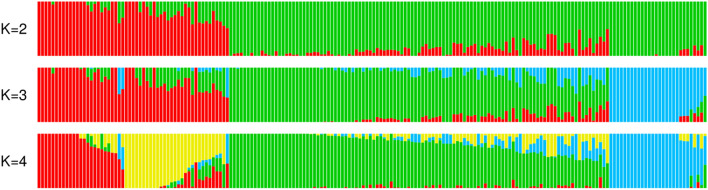
Population structure constructed using the SNPs data for the individual ancestry estimated using the ADMIXTURE analysis. Individuals are represented in thin vertical lines separated into segments corresponding to the assumed membership in K = 2, 3 and 4 genetic groups as shown by colors.Each color represents one ancestral subpopulation.

The SNP calling analysis revealed 10,794 high-quality SNPs among the studied faba genotypes. The sequence variations of these SNPs were C/T (4251 SNPs), and A/G (4029 SNPs), followed by A/T (836 SNPs), G/T (761 SNPs), A/C (619 SNPs), and C/G (298 SNPs). The average CV values for the 100 replicates of the population structure started to increase directly after K = 2 indicating the presence of two ancestral subpopulations in the germplasm set used in the present study. However, we presented the results of K up to 4 because their 100 replicate runs resulted in comparable classification of genotypes into ancestral subpopulations with top > 20% of replicates having almost the exact log-likelihood values (Fig. 3). Beyond K = 4, the analysis started to output arbitrary results with inconsistent classifications even for the top 10 replicates with the highest log-likelihood values.

### GWAS and annotation analyses

As we ended with a total of 10,794 high-quality SNPs after filtration, the Bonferroni significant threshold can be calculated as (0.05/10,794 = 4.6E−6). Analyzing all 103 traits (including RI scores) with the ST-GWAS model resulted in only 10 highly significant associations with the Bonferroni threshold as well as 110 suggestive associations for only 66 traits while the remaining traits had no associated SNPs (Supplementary Table S1). These associations were represented by 105 SNPs. Only one SNP (*SNODE_27970_52*) for DFLR was associated with three treatments (I, M and C) in TR16, while another five SNPs were associated with two scores for PLHT or DFLR (Supplementary Table S1). Of these, two SNPs (*SNODE_168698_34* for PLHT with treatment I, and *SNODE_23759_68* for DFLR with treatment M in TR16) were associated with a specific trait and its correspondence RI score. Another five SNPs showed association with two different traits of which *SNODE_3696_16* and *SNODE_77186_51* were associated with GYPPLT in TR16, treatments M and I respectively, while *SNODE_22383_32* was associated with RI in TR16, treatment M, for the same traits. *SNODE_239220_75* was associated with TR19_DMAT_I and TR16_NPPT_I_RI, while *SNODE_7114_58* showed associations with five DFLR and DMAT across environments/treatments (Supplementary Table S1).

The MT-GWAS model for 20 traits across environments (including RI scores) resulted in 14 highly significant associations and 64 suggestive associations for all traits represented by 72 SNPs (Table 3). The largest number of associations (12) were detected of DMAT, while GYPPLT_RI, NPPT_RI and Score1 had the lowest number with only one association each. Most of the SNPs that showed associations with multiple traits/treatments in the ST-GWAS analysis were also detected in MT-GWAS analysis. Four SNPs showed associations with a specific trait with its reduction index which were *SNODE_23759_68* for DFLR, *SNODE_14558_21* for NSPP, *SCONTIG73439_18* for PLHT, and *SNODE_103_72* for NBBR (Table 3). The SNP *SCONTIG127798_41* was associated with GCC and DFLR while the SNP *SNODE_22383_32* was associated with GYPPLT (Table 3).

**Table 3:** SNP-trait associations revealed by the MT-GWAS analysis.

Trait	SNP	allele1	allele0	MAF	P
DFLR	SNODE_7114_58	C	T	0.14	3.0E−07
DFLR	SNODE_162178_22	A	G	0.07	9.8E−07
DFLR	SNODE_27970_52	C	G	0.17	3.6E−05
DFLR	***SNODE_23759_68***	A	G	0.11	7.1E−05
DFLR_RI	***SNODE_23759_68***	A	G	0.10	8.6E−07
DFLR_RI	SNODE_5725_31	C	T	0.41	3.2E−06
DFLR_RI	SNODE_4187_38	G	A	0.05	3.3E−06
DFLR_RI	SNODE_26501_64	G	C	0.13	9.5E−06
DFLR_RI	***SCONTIG127798_41***	C	T	0.07	2.9E−05
DFLR_RI	SNODE_1051_18	C	T	0.08	4.3E−05
DMAT	SCONTIG72526_35	A	G	0.05	4.6E−06
DMAT	SNODE_375879_34	A	T	0.08	5.7E−06
DMAT	SCONTIG93616_28	C	T	0.30	1.3E−05
DMAT	SCONTIG6418_84	C	T	0.22	1.4E−05
DMAT	SNODE_61301_40	T	C	0.06	1.5E−05
DMAT	SNODE_80758_20	C	T	0.06	3.3E−05
DMAT	SNODE_483217_44	G	A	0.12	4.6E−05
DMAT	SNODE_143506_34	G	A	0.05	6.9E−05
DMAT	SCONTIG125372_89	T	C	0.05	8.3E−05
DMAT	SNODE_6229_36	C	T	0.11	8.4E−05
DMAT	SCONTIG79953_82	T	C	0.37	8.6E−05
DMAT	SNODE_16244_35	T	C	0.33	8.8E−05
DMAT_RI	SNODE_76542_45	T	G	0.06	5.1E−06
DMAT_RI	SNODE_13235_37	C	A	0.25	2.5E−05
GCC	SNODE_11304_24	A	T	0.31	3.5E−06
GCC	SCONTIG24931_19	G	A	0.06	4.4E−05
GCC	SCONTIG66488_16	G	A	0.07	7.8E−05
GCC_RI	***SCONTIG127798_41***	C	T	0.07	5.3E−08
GCC_RI	SCONTIG75553_52	T	C	0.34	8.3E−05
GCC_RI	SNODE_12134_67	T	G	0.07	9.3E−05
GYPLT	SNODE_77186_51	T	A	0.10	1.3E−05
GYPLT	SNODE_3696_16	G	A	0.11	2.5E−05
GYPLT	SNODE_54972_30	A	C	0.14	1.3E−07
GYPLT	SCONTIG46666_46	A	T	0.06	2.6E−06
GYPLT	SNODE_167460_49	C	T	0.20	6.7E−06
GYPLT	SCONTIG90061_39	A	C	0.06	8.1E−06
GYPLT	SNODE_5674_14	G	A	0.06	1.1E−05
GYPLT	SNODE_4555_43	T	A	0.11	3.8E−05
GYPLT	SNODE_34407_21	A	G	0.06	5.5E−05
GYPLT	SNODE_4363_81	C	T	0.36	7.5E−05
GYPLT	SNODE_16972_9	A	G	0.05	7.9E−05
GYPLT _RI	***SNODE_22383_32***	T	C	0.14	5.5E−05
NBrPLT	SCONTIG97891_72	A	G	0.40	7.4E−06
NBrPLT	***SNODE_103_72***	T	C	0.25	2.6E−05
NBrPLT_RI	SNODE_173108_18	A	G	0.06	2.6E−06
NBrPLT_RI	SNODE_2942_50	C	T	0.06	1.0E−05
NBrPLT_RI	***SNODE_103_72***	T	C	0.25	1.6E−05
NBrPLT_RI	SNODE_144193_69	C	A	0.46	9.5E−05
NPPLT	SCONTIG23347_118	G	A	0.07	2.3E−05
NPPLT	SNODE_28265_65	C	T	0.31	8.0E−05
NPPLT_RI	SNODE_559376_60	A	T	0.45	1.0E−11
NSPLT	***SNODE_14558_21***	A	G	0.20	1.2E−05
NSPLT	SCONTIG38056_40	C	T	0.07	2.8E−05
PLHT	SNODE_43134_109	A	G	0.05	2.8E−09
PLHT	SNODE_134600_32	T	G	0.06	3.8E−06
PLHT	SNODE_27201_27	T	G	0.06	1.0E−05
PLHT	***SCONTIG73439_18***	G	A	0.06	1.3E−05
PLHT	SNODE_78412_27	T	C	0.11	2.9E−05
PLHT	SNODE_124581_38	A	C	0.07	6.9E−05
PLHT	SNODE_113123_17	T	G	0.06	7.5E−05
PLHT	SCONTIG57859_65	G	A	0.07	7.5E−05
PLHT	SCONTIG101530_33	A	G	0.14	9.6E−05
PLHT_RI	***SCONTIG73439_18***	G	A	0.06	6.1E−06
PLHT_RI	SCONTIG157_70	T	G	0.16	9.3E−06
PLHT_RI	SNODE_14298_44	A	G	0.06	1.0E−05
PLHT_RI	SNODE_3358_54	A	C	0.18	1.1E−05
PLHT_RI	SNODE_11304_26	G	A	0.12	2.9E−05
PLHT_RI	SNODE_107804_70	A	T	0.05	3.2E−05
PLHT_RI	SNODE_176979_47	G	A	0.08	4.6E−05
PLHT_RI	SNODE_4904_26	C	T	0.17	9.2E−05
HDS1	SNODE_2908_40	G	A	0.17	4.4E−05
HDS2	SNODE_8269_115	G	A	0.07	5.8E−06
HDS2	SNODE_68619_39	G	A	0.08	1.3E−05
HDS2	SNODE_2018_104	C	T	0.07	9.9E−05
NSPLT_RI	SNODE_2107_36	C	T	0.06	6.1E−06
NSPLT_RI	SNODE_7966_59	G	A	0.06	4.2E−05
NSPLT_RI	***SNODE_14558_21***	A	G	0.20	8.7E−05

Underscored SNPs represents the highly significant associations, while SNPs in bold italic represents the SNPs associated with multiple traits.

*SNP* single nucleotide polymorphism, *MAF* minor allele frequency, *DFLR* days to flowering, *DFLR_RI* DFLR reduction index, *DMAT* days to maturity, *DMAT_RI* DMAT reduction index, *GCC* green canopy cover, *GCC_RI* green canopy cover reduction index, *GYPLT* grain yield per plant, *GYPLT_RI* GYPLT reduction index, *NBrPLT* number of branches per plant, *NBrPLT_RI* NBrPLT reduction index, *NPPLT* number of pods per plant, *NPPLT_RI* NPPLT resuction index, *NSPLT* number of seeds per plant, *PLHT* plant height, *PLHT_RI* reduction index of PLHT, *HDS1* first herbicide damage score, *HDS2* second herbicide damage score, *NSPLT_RI* number of seeds per plant, *NSPLT* number of seeds per plant, _ reduction index.

Gene annotation showed that SNP *SCONTIG127798_41* associated with reduction index of GCC and DFLR is located within a gene annotated as acidic endo-chitinase annotation, *SNODE_14298_44* associated with the reduction index of PLHT is located within a gene annotated as LRR receptor-like serine/threonine-protein kinase RCH1, *SNODE_3696_16* associated with GYPLT is located within a gene annotated as Probable serine/threonine-protein kinase NAK, *SNODE_4187_38* associated with the reduction index of DFLR is located within a gene annotated as malate dehydrogenase, *SNODE_559376_60* associated with the reduction index of NPPLT is located within a gene annotated as photosystem I core protein PsaA, while *SNODE_7114_58* associated with DFLR is located within a gene annotated as MYB-related protein P-like (Supplementary Table S2).

## Discussion

Weed menace is a serious threat to faba bean production, and the identification of herbicide-tolerant varieties is one of the most effective methods for weed control. The results obtained from the present field and greenhouse studies demonstrated how the post-emergence application of metribuzin or imazethapyr negatively affects faba bean plants. Herbicide application affected the crop phenology by delaying flowering and maturity. Although the delayed flowering helps plant escape the risk of frost in regions like Western Australia, there might be a potential yield penalty as the plants run out of moisture before it can fill its grain^43,44^. In addition, herbicide application also affected biological and grain yields of faba bean by reducing plant height, green canopy cover, and grain yield components and by increasing the number of branches. Many studies^30,42,45–48^ reported significant reduction in plant height, grain yield and yield components while studying the effect of post-emergence herbicide application on faba bean, lentil and chickpea. On the other hand, Wall^49^ and Sajja et al.^50^, reported an increase in the number of branches of treated plants. The observed damage after metribuzin and imazethapyr treatments is the consequence of the growth inhibition caused by both herbicides. Metribuzin hampers photosynthesis activity by inhibiting the photosynthetic electron flow^51,52^ and imazethapyr inhibits acetolactate synthase (ALS)^53^, the first common enzyme in the biosynthesis of the branched-chain amino acids^54^ causing the death of meristematic cells. On the other hand, significant increase in the number of branches in herbicide treated plants could be caused by the plant recovery which occurs at the lateral meristem in dicots resulting in the development of new branches. The genotypic variation observed in the herbicide damage scores (HDS) highlights the difference in the reaction of each genotype toward post emergence herbicide application in faba bean. This observation was expected as the evaluated genotypes are genetically diverse^22^. The differences observed between the first (HDS1) and the second (HDS2) scores were due to the recovery or deterioration of the plants one month after herbicide treatment. The recovery might result from the metabolism of the herbicides into inactive compounds^55^. Therefore, the observed differences in the genotype ability to recover might be due to differential rate of metabolic degradation for imazethapyr treatment^47^ and to differential disruption of electron transfer for metribuzin treatment.

Population structure analysis revealed two major ancestral populations for the germplasm which is compatible with the original germplasm of 995 genotypes genotyped with 20 microsatellite markers, from which this population was selected^22 **22**^. As expected, MT-GWAS analysis exposed higher detection power compared to ST-GWAS analysis due to the larger datapoint fitted in the model which is equivalent to increasing the population size^21^. This was revealed by the larger number of highly significant as well as suggestive association per trait detected (Table 3, Supplementary Table S1). Another advantage is the ability to detect QTL with stable effect across different environments or treatments which should have higher potential to improve the efficiency of marker assisted selection in diverse environments^56,57^.

To the best of our knowledge, the present study is the first GWAS for herbicide tolerance in faba bean and the first for all phenotyped traits under the control treatment with no herbicide application. Thus, most of the QTL detected in the present study seem novel and have not been reported before. Very limited studies used SNP data on biparental or multi-parental faba bean populations^15,26,58^ but none aimed to dissect quantitative traits in natural diverse populations. Sallam and Martsch^58^ associated 156 SNPs with frost tolerance in a population derived from 11 parental lines, while Ali et al.^59^ used the same population to detect loci associated with freezing and drought tolerance using 175 SNPs and AFLP markers. The identification of QTL through GWAS in faba bean is complex due to the large undecoded genome and highly repetitive sequences. These issues have delayed the progress made towards the development of genomic resources and marker assisted selection in faba bean breeding programs^60^.

Identification of key genes, mechanisms and functional markers is essential to develop herbicide tolerant faba beans. The associations between some genes identified in this study and herbicide tolerance have been reported previously in different crops. Acidic endochitinase and malate dehydrogenase which were found to be associated with the reduction indexes of DFLR and GCC were among the proteins affected by the application of sulfonylurea herbicide in soybeans^61^. Sulfonylurea herbicides and imazethapyr have similar mode of action; both herbicides block the biosynthesis of the branched-chain amino acids^54,62^. The two protein kinase LRR receptor-like serine/threonine-protein kinase RCH1 and probable serine/threonine-protein kinase NAK which were found to be associated with the reduction index of PLHT and GYPLT in the present study are generally considered key regulators of plant architecture and growth behavior, and the expansion of these proteins during plant evolution has also been correlated with the specific adaptations of the species in defense and stress responses^63^. Their direct involvement in abiotic stress resistance (drought, heat, cold, salinity) has also been demonstrated in different studies^64–66^. Burns et al.^67^ concluded that herbicide stress is perceived similarly to other abiotic stresses and reported modification in the level of the protein kinase gene family in the multiple herbicide resistant *Avena fatua*.

The MYB-related protein P-like which was found associated with DFLR is involved in herbicide tolerance belongs to the *MYB* gene family that comprises one of the richest groups of transcription factors in plants. Members of this family have a well-established role in abiotic stress responses^68,69^. Bhoite et al.^70^ found also that the transcription factors MYB were significantly expressed under metribuzin stress. The photosystem I (PS I) core protein PsaA that is found in the present study to be associated with the reduction index of NPPLT is a subunit membrane protein complex involved in photosynthesis. PS I and PS II drive the light reaction of photosynthesis. The first stage of the light reaction occurs in PS II whereas the final stage of the light reaction occurs in PS I^71^. The metribuzin applied to faba bean plants in this study inhibits PS II by disrupting electron transfer through binding to the D1 protein of the photosystem II complex in chloroplast thylakoid membranes^51^. This mode of action explains the involvement of the PS I in the reaction toward herbicide application especially that PS II comes first in the path of the electron flow followed by PS I.

The described mechanism of action of the annotated genes suggests that DFLR_RI and GCC_RI are associated with tolerance to imazethapyr while DFLR and NPPLT_RI are associated with tolerance to metribuzin, and GYPLT and PLHT_RI are associated with tolerance to both herbicides.

## Conclusions

Weeds represent a major problem to faba bean crop which limits its expansion in many production regions. By excluding faba bean and other legume from the cropping system, cereal monoculture will continue to deplete the soil, lowering its quality and indirectly reducing yield and quality of the produce. Herbicide tolerant faba bean lines could be a game changer in the reintegration of faba bean in modern cropping systems as it contributes to the reduction of production cost by avoiding excessive use of manual weeding. Considering the many advantages of herbicide tolerance in faba bean, it is imperative to breed elite cultivars that features this trait. However, field selection is very costly and time consuming. The integration of genomic selection and marker assisted selection will improve selection accuracy, increase the selection intensity and shorten the breeding cycle when selecting at early generations. In the present study, we identified genomic regions associated with tolerance to imazethapyr and metribuzin herbicides as highly significant associations between SNPs markers and phenological and yield traits related to herbicide tolerance were detected using multi-trait association. These markers will be useful for improving the efficiency of faba bean programs and represent important steps towards the selections for herbicide tolerance.

## Materials and methods

### Plant materials

A set of 134 faba bean genotypes comprising 118 landraces from 42 countries and 16 ICARDA breeding lines that were used to establish a reference set under the Generation Challenge Program (GCP) was used for phenotyping and genotyping in the present study. Previous assessment with Simple Sequence Repeat (SSR) markers showed that the set was genetically diverse and comprised 45 *major,* 17 *minor,* 63 *equina* and 9 *paucijuga* genotypes^22,42^. In addition to the test genotypes, a total of 6 faba bean cultivars (FLIP86-98, ILB1814, Ed-damer, Hudeiba-93, Shambat-75, SML) were included in the experiments. The seeds used in the current experiments are sourced from the reserve seeds that are multiplied each year under insect-proof cages in order to ensure purity of the evaluated accessions.

### Experiments

A total of four experiments were conducted: three field and one greenhouse experiments.

#### Field experiments

A total of three field experiments were conducted at two ICARDA research stations: Marchouch (33.558°N 6.693°W, altitude 255 m) in Morocco and Terbol (35.98°N, 33.88°E, altitude 890 m) in the Bekaa Valley of Lebanon. Marchouch station is characterized by the semi-arid environment with a Vertisol soil, mostly silty clay, while Terbol station is characterized by cool and high rainfall winter and moderate wet spring with a deep and rich clay loam soil. Each experiment comprised three treatments applied at the pre-flowering stage : T1-Metribuzin @250 g ai/ha, T2-Imazethapyr @ 75 g ai/ha and T3- No herbicide application. Faba bean genotypes were sown in rotation with cereals in mid-December at Marchouch 2014/2015, late November at Terbol 2014/2015 and 2018/2019 main seasons. Each genotype was planted in 2 m long two-row plot with 0.5 m spacing between rows. At Marchouch, the crop received 291 mm of precipitation during the cropping season in addition to 30 mm irrigation during early vegetative phase; the crop was exposed to intermittent drought and heat. 120 genotypes along with the three following cultivars FLIP86-98,ILB1814 and Hudeiba-93were evaluated at Marchouch using Augmented design^42^. At Terbol, a total precipitation of 343 mm and 810 mm was recorded respectively during 2015/2016 and 2018/2019 cropping seasons. Supplemental irrigation (30 mm) was provided at Terbol station in 2015/2016 season during dry-spell periods, while no irrigation was provided in case of highly and well distributed rains in 2018/2019. A total of 134 genotypes were evaluated at Terbol using Alpha lattice design. In 2015/2016 season, the field experiment was conducted with two replicates and 15 blocks and with the cultivar FLIP86-98^42^ and in 2018/2019 the field experiment was conducted with 3 replicates and 14 blocks and the following cultivars FLIP86-98, ILB1814, Ed-damer, Hudeiba-93,Shambat-75 and SML.

#### Greenhouse experiment

The germplasm genotypes along with six checks (FLIP86-98, ILB1814, Ed-damer, Hudeiba-93, Shambat-75, SML) were evaluated in an alpha design with two replicates and two treatments: 250 g ai/ha of metribuzin and untreated treatment during 2017/2018 cropping season. Three seeds per pot for each genotype were sown in this experiment. Irrigation was provided regularly to maintain 100% soil water capacity in pots. Temperature inside the greenhouse was fixed at 24 to 28 °C the optimal day time temperature of faba bean.

The herbicide treatments applied in all experiments are metribuzin (M, T1), imazethapyr (I, T2) and the control treatment (C, T3) in which no herbicide was applied. The doses of herbicides applied are the recommended doses as per the labels of metribuzin (Sencor: Bayer) and imazethapyr (Pursuit: BASF). Both herbicides were uniformly sprayed at the rate of 250 g ai ha^−1^ and 75 g ai ha^−1^ respectively at the inflorescence stage BBCH code 5072^72^ for the field experiments and at the stem elongation stage BBCH code 30^72^ for the greenhouse experiment using an electric sprayer with automated flow (375 L/ha). In the field, the herbicide was sprayed early in the morning to ensure a low wind speed. Details of traits scored in each trial can be found in Supplementary Table S3. Traits were coded as the environment, followed by the trait, the treatment and “RI” if the score describe a reduction index. For the multi-trait GWAS analysis, the trait name does not have the name of the environment or the treatment.

### Phenotyping for herbicide tolerance

Observations (Supplementary Table S3) were recorded on days to 50% flowering (DFLR) and maturity (DMAT) on plot basis for the untreated treatment, and plant height (PLHT) and grain yield per plant (GYPLT) on three plants selected randomly for all the three treatments at Marchouch 2014/2015. At Terbol station, the following additional traits were also recorded on three plants selected randomly for the three treatments: number of pods per plant (NPPLT), number of seeds per plant (NSPLT), number of branches per plant (NBrPLT) and green canopy cover (GCC). Green canopy cover expressed as the average percentage of green coverage of three plants was quantified using the Canopeo application developed by Oklahoma State University using Matlab. Under the greenhouse conditions where temperature was controlled at optimal conditions, PLHT was recorded at flowering PLHT_1 (BBCH code 60) and pod development PLHT_2 (BBCH code 70) stages^72^. The herbicide damage score (HDS) was recorded in all the four experiments using a 1-5 scale^42^ (Supplementary Table S4) at flowering (HDS1) and pod development (HDS2) stages. The ratio of each quantitative trait was calculated for each plot using the following formula described by Abou-Khater et al.^42^:

$$RI\% = 100 - \left( {\frac{T}{C} \times 100} \right)$$

where RI%, the reduction index, represents the reduction or penalty in traits of herbicides treated plots compared to the control untreated plots, *T́* is the average of plots treated with herbicide (metribuzin or imazethapyr); *Ć* is the mean of genotypes under untreated conditions.

### DNA extraction and genome by sequencing analysis

Genomic DNA was extracted from young leaf tissues for each tested genotype using the DNeasy 96 Plant Kit (QIAGEN, Valencia, CA, USA) Qiagen Plant DNA Preparation Kit. For the preparation of the GBS library, the two restriction enzymes, *PstI and MspI,* were used to generate fewer variation in the distribution of read depth and higher number of scorable SNPs. GBS libraries were prepared with 48 barcode adapters with 4–9 bp sequence^73^. The single read (100 base pairs) sequencing on an Illumina HiSeq 2500 produced approximately 4 million reads per genotype. Raw read sequences were processed using TASSEL-GBS 5.0 with the default parameters^74^. A faba bean sequence database was constructed using 223,801 genomic and transcriptomic faba bean sequences downloaded from NCBI and pulsedb databases (www.ncbi.nlm.nih.gov and https://www.pulsedb.org/analysis/136) and additional faba bean sequences constructed using the Trinity assembler from one run of the GBS files. These sequences were used as a reference to align GBS sequence tags and indexed using Bowtie2 version 2.2.4^75^ Bowtie2 was used to align GBS tags to faba sequences using the–very-sensitive-local option. Resulting SNPs were filtered with 20x coverage, where SNPs with more than 15% missing data or less than 5% minor allele frequency (MAF) were removed. SNPs were named by contig base pair position.

### Statistical analysis of phenotyping data

The spatial statistical model was applied for variance analysis for all quantitative data using the Automatic Spatial Analysis of Row-Column modules of Genstat 19 edition^76^. Significance of variation among genotypes and treatments was assessed in terms of *P*-values. The analysis of variance (ANOVA), means of genotypes, means of treatments and interactions between genotypes and treatments were estimated with standard errors using best linear unbiased prediction (BLUP) values using GenStat software. BLUPs were used to conduct all downstream analyses. Multi environment trials analysis (META) were conducted to evaluate variation among genotypes, treatments and the genotype × treatment interaction across trials for the traits recorded in more than one trial. Genotype and treatment were fitted as fixed parameters while environment (year-location) were fitted as random parameter.

### Genome-wide association analysis

ADMIXTURE software^77^ was used to infer population structure with the number of underlying subpopulations (K) ranges between 2 and 20. The analysis was run with 100 random replicates and 20 cross validations. The most probable K was determined at the point when the average cross validation (CV) values across the 100 replicates started to increase. Single-trait (ST) and Multi-trait (MT) GWAS was fitted using GEMMA software^21^ by fitting each trait independently (for the ST analysis) or fitting all field or greenhouse records together (for the MT analysis) with the default parameters and by fitting the genomic relatedness matrix as a covariate to control for population stratification^78^. Bonferroni correction was used to determine the significant threshold at p < 0.05 but all SNPs with p < 1E−4 were presented as suggestive associations. Pairwise linkage disequilibrium (LD) between associated SNPs within each trait was estimated with the r2 statistics following Weir^79^ to determine the SNPs that are associated with the same quantitative trait locus (QTL). Genomic sequences containing herbicide tolerance associated SNPs were aligned against the NCBI database using BLASTX tool using default parameters to annotate potential candidate genes underlying the causal variants.

### Ethcial approval

The authors confirm that the study complies with local and national regulations. The seeds were collected from the genebank of the International Center for Agricultural Research in the Dry Areas (ICARDA) for research purposes according the International Treaty of Plant Genetic Resources for Food and Agriculture (ITPGRFA). For the collection of seeds, all relevant permits or permissions have been obtained.The seeds flow from ICARDA GenBank at Terbol to Morocco was made following the phytosanitary regulations of both countries and using the Standard Material Transfer Agreement (SMTA) governed by ITPGRFA. The experiments were conducted at ICARDA sites at Terbol and Marchouch in accordance to National and International regulations.

## Supplementary Information

Supplementary Information.

## Data Availability

The datasets generated and analyzed during the current study are available from the corresponding authors on request.
