# Supplementary material for: Genomic regions associated with herbicide tolerance in a worldwide faba bean (Vicia faba L.) collection
Source: Sci Rep. 2022 Jan 7;12:158. doi: 10.1038/s41598-021-03861-0 (PMC8741826; doi:10.1038/s41598-021-03861-0)
Supplement: Supplementary file 1 — Supplementary Information. [file 41598_2021_3861_MOESM1_ESM.pdf]

Genomic regions associated with herbicide tolerance in a worldwide faba bean (*Vicia faba* L.) collection

Lynn Abou-Khater[1*], Fouad Maalouf[1], Abdulqader Jighly[5], Alsamman M Alsamman[6], Diego Rubiales[4], Nicolas Rispail[4], Jinguo Hu[7], Yu Ma[8], Rind Balech[1], Aladdin Hamwieh[3], Michael Baum[2] and Shiv Kumar[2]**

[1] International Center for Agricultural Research in the Dry Areas (ICARDA), Terbol, Lebanon.
[2] ICARDA, Rabat, Morocco.
[3] ICARDA, Cairo, Egypt.
[4] Institute for Sustainable Agriculture, CSIC, 14004 Córdoba, Spain.
[5] Agriculture Victoria, Victoria, Australia.
[6] Agricultural Genetic Engineering Research Institute, Cairo, Egypt.
[7] USDA-ARS Plant Germplasm Introduction & Testing Research Unit, Pullman, USA.
[8] Department of Horticulture, Washington State University, Pullman, USA

Corresponding authors e-mail: [*]lynnaboukhater@hotmail.com; [**]f.maalouf@cgiar.org.

Supplementary Table S1. SNP-trait associations revealed by the ST-GWAS analysis. Underscored SNPs represents the highly significant associations, while SNPs in italic represents the SNPs associated with multiple traits

Trait	SNP	allele1	allele0	MAF	Effect	P	-Log10
TR16_DFLRI_RI	SNODE_5725_31	C	T	0.41	5.66	4.3E-11	10.4
TR16_DFLRI_RI	SCONTIG127798_41	C	T	0.07	-2.60	5.0E-11	10.3
TR16_DFLRM_RI	***SNODE_23759_68***	A	G	0.10	6.67	2.1E-07	6.7
MR15_PLTHTI	SNODE_106460_36	T	C	0.05	18.29	4.3E-07	6.4
TRS18_HDS1M	SNODE_13467_45	A	G	0.19	0.70	1.1E-06	6.0
MR15_GYPLTI	SNODE_4555_43	T	A	0.10	12.18	1.5E-06	5.8
TR16_DFLRI	***SNODE_7114_58***	C	T	0.11	6.71	2.5E-06	5.6
TR19_NBrPLTM_RI	SNODE_173108_18	A	G	0.06	65.35	3.0E-06	5.5
TRS18_GCCC	SNODE_12390_16	C	T	0.21	1.27	3.0E-06	5.5
TR16_DFLRM_RI	***SNODE_4187_38***	G	A	0.05	7.81	3.3E-06	5.5
TR16_NSPLTM	SCONTIG38056_40	C	T	0.07	9.44	5.9E-06	5.2
TR19_NPPLTI	SCONTIG16487_99	T	C	0.07	5.53	6.1E-06	5.2
TR16_DMATI	SNODE_76542_45	T	G	0.05	-3.24	7.0E-06	5.2
MR15_PLTHTM_RI	***SNODE_14298_44***	A	G	0.06	63.37	7.1E-06	5.1
TR16_GYPLTI	***SNODE_77186_51***	T	A	0.10	7.97	7.2E-06	5.1
MR15_PLTHTI_RI	SCONTIG65312_44	T	C	0.05	89.46	9.1E-06	5.0
MR15_PLTHTI_RI	SNODE_783_18	G	A	0.06	83.04	1.0E-05	5.0
TR19_NBrPLTM_RI	SNODE_2942_50	C	T	0.06	61.37	1.0E-05	5.0
TR19_NBrPLTI_RI	SNODE_103_72	T	C	0.25	27.28	1.1E-05	5.0
TR16_DFLRI_RI	SCONTIG5600_39	C	T	0.06	7.01	1.2E-05	4.9
TR16_DFLRM	***SNODE_7114_58***	C	T	0.11	6.69	1.2E-05	4.9
TR19_GCCC	SNODE_46550_53	C	A	0.41	5.17	1.5E-05	4.8
TRS18_HDS2M	SNODE_132611_47	T	G	0.06	1.48	1.5E-05	4.8
TR16_NPPLTTI_RI	SNODE_522694_66	T	C	0.07	53.48	1.6E-05	4.8
TR19_GCCM_RI	SNODE_18326_71	G	A	0.37	14.61	1.6E-05	4.8
MR15_GYPLTM	SNODE_32018_65	A	G	0.20	6.33	1.6E-05	4.8
TR19_GYPLTI	SNODE_4363_81	C	T	0.34	4.00	1.7E-05	4.8
TRS18_HDS1M	SCONTIG65425_78	T	C	0.22	0.52	1.7E-05	4.8

TR16_DMATI	SNODE_5210_41	G	A	0.24	-1.31	1.8E-05	4.7
TR16_DMATI	SNODE_5352_33	C	T	0.43	-1.23	1.9E-05	4.7
MR15_PLTHTI_RI	***SNODE_303749_26***	G	C	0.09	55.58	1.9E-05	4.7
TR16_PLHTI_RI	***SNODE_168698_34***	G	A	0.31	-7.68	2.0E-05	4.7
TR19_DMATM	SNODE_1938_61	T	G	0.10	0.91	2.1E-05	4.7
TR16_DMATC	***SNODE_7114_58***	C	T	0.11	1.67	2.2E-05	4.7
TR19_DMATC	SNODE_176062_39	G	T	0.39	0.66	2.2E-05	4.7
TR16_PLHTM	SNODE_1327_40	G	A	0.26	-4.92	2.2E-05	4.7
MR15_PLTHTI_RI	SCONTIG16540_74	G	A	0.06	78.04	2.3E-05	4.6
MR15_GYPLTI	SNODE_49416_94	A	G	0.07	12.46	2.4E-05	4.6
TR19_DMATI	SCONTIG93219_127	A	C	0.05	2.09	2.5E-05	4.6
TR19_NPPLTC	SNODE_159494_68	G	A	0.07	6.96	2.7E-05	4.6
MR15_PLTHTI	SNODE_4025_42	T	G	0.06	16.12	2.7E-05	4.6
MR15_PLTHTI_RI	SNODE_11304_26	G	A	0.13	44.41	2.8E-05	4.6
MR15_PLTHTI_RI	***SNODE_14298_44***	A	G	0.06	58.04	2.8E-05	4.6
TR19_NSPI	SNODE_99859_41	C	A	0.05	11.79	2.8E-05	4.5
MR15_GYPLTM	SCONTIG8042_30	T	G	0.14	9.13	2.9E-05	4.5
TR16_DFLRI	***SNODE_27970_52***	C	G	0.17	4.79	3.0E-05	4.5
TRS18_PLHT2C	SNODE_8117_82	T	C	0.41	-3.63	3.0E-05	4.5
TR16_DMATI	SNODE_5497_184	C	T	0.07	-2.62	3.1E-05	4.5
TR16_NPPLTTM_RI	SNODE_13896_44	T	G	0.21	33.37	3.1E-05	4.5
MR15_GYPLTM	SCONTIG124093_52	T	A	0.06	14.06	3.1E-05	4.5
MR15_PLTHTM_RI	SNODE_154943_14	T	C	0.08	52.28	3.4E-05	4.5
MR15_GYPLTI	SNODE_113699_29	T	C	0.17	8.18	3.5E-05	4.5
TR19_ScoreI	SNODE_6947_50	A	G	0.22	0.15	3.6E-05	4.4
TR16_GYPLTM_RI	***SNODE_22383_32***	T	C	0.14	23.39	3.6E-05	4.4
TR16_DFLRI_RI	SNODE_12919_43	A	T	0.11	3.97	3.6E-05	4.4
TR16_DMATM	SCONTIG38602_68	G	A	0.06	-2.68	3.7E-05	4.4
TR19_DFLRM	SNODE_73156_58	C	T	0.19	4.17	3.7E-05	4.4
TR19_NPPLTI	SNODE_13244_37	C	T	0.08	4.84	3.7E-05	4.4
TR16_GYPLTM	***SNODE_3696_16***	G	A	0.10	62.71	3.9E-05	4.4
TRS18_GCCC	SNODE_11304_24	A	T	0.31	-0.89	4.1E-05	4.4
TR19_ScoreI	SCONTIG57859_65	G	A	0.07	-0.31	4.2E-05	4.4
TR19_DMATI	***SNODE_239220_75***	G	C	0.41	1.03	4.3E-05	4.4
MR15_PLTHTM_RI	***SNODE_303749_26***	G	C	0.09	54.27	4.3E-05	4.4
TR19_NBrPLTM_RI	SNODE_144193_69	C	A	0.46	-66.48	4.4E-05	4.4
MR15_GYPLTI	SCONTIG90061_39	A	C	0.06	13.39	4.4E-05	4.4
TR16_GYPLTM	***SNODE_3696_16***	G	A	0.10	5.23	4.5E-05	4.3
TRS18_PLHT2M	SNODE_50475_21	A	G	0.23	3.85	4.5E-05	4.3
TR16_DFLRM	***SNODE_27970_52***	C	G	0.16	5.02	4.6E-05	4.3
TR16_DFLRM_RI	SNODE_1051_18	C	T	0.08	5.93	4.8E-05	4.3
TR16_PLHTC	SCONTIG124142_38	A	G	0.11	-7.42	5.0E-05	4.3
TR16_HDS1I	SCONTIG124448_24	G	A	0.47	0.91	5.0E-05	4.3
MR15_PLTHTI_RI	SCONTIG86606_73	G	A	0.07	75.29	5.1E-05	4.3
TR19_NBrPLTC	SCONTIG97891_72	A	G	0.40	0.64	5.3E-05	4.3
TR19_GCCI_RI	SNODE_8714_56	G	A	0.12	26.15	5.4E-05	4.3

MR15_GYPLTC	SCONTIG107603_117	G	A	0.33	8.52	5.5E-05	4.3
TR16_DFLRM	***SNODE_23759_68***	A	G	0.10	8.39	5.6E-05	4.3
TR16_DFLRC	***SNODE_7114_58***	C	T	0.11	3.49	5.6E-05	4.3
TR16_NSPLTI	SNODE_27984_41	T	C	0.09	-6.51	5.6E-05	4.2
TRS18_GCCM_RI	SNODE_6438_25	T	A	0.28	23.95	5.7E-05	4.2
TR19_NBrPLTC	SNODE_1799_57	A	T	0.16	0.52	5.7E-05	4.2
TR16_GYPLTM_RI	***SNODE_22383_32***	T	C	0.14	21.58	5.9E-05	4.2
MR15_PLTHTI_RI	SNODE_6940_56	C	T	0.13	42.12	5.9E-05	4.2
TR19_NSPM	SNODE_784518_64	C	T	0.43	-4.05	6.0E-05	4.2
TR16_DFLRM_RI	SCONTIG20800_23	G	A	0.06	6.56	6.0E-05	4.2
MR15_PLTHTI_RI	SNODE_2199_68	G	A	0.07	68.70	6.2E-05	4.2
TR16_PLHTI	***SNODE_168698_34***	G	A	0.31	-5.44	6.2E-05	4.2
TR16_NPPLTTM_RI	SNODE_55885_28	C	T	0.09	47.66	6.3E-05	4.2
TR19_DMATI_RI	SNODE_13235_37	C	A	0.25	-0.43	6.3E-05	4.2
TR16_PLHTM_RI	SCONTIG99169_68	C	T	0.05	-12.13	6.6E-05	4.2
MR15_GYPLTI	SCONTIG46666_46	A	T	0.07	12.22	6.7E-05	4.2
TR16_DFLRI_RI	***SNODE_4187_38***	G	A	0.05	6.82	6.8E-05	4.2
MR15_PLTHTI_RI	SNODE_34274_45	A	G	0.11	46.06	7.0E-05	4.2
TR16_NPPLTTI_RI	SNODE_503024_18	T	A	0.40	23.17	7.3E-05	4.1
TR16_NSPLTC	SNODE_125479_23	A	T	0.28	5.86	7.3E-05	4.1
TR16_DFLRC	SNODE_9493_68	T	C	0.06	4.19	7.3E-05	4.1
TR16_DFLRM_RI	SNODE_8481_51	T	C	0.17	3.50	7.4E-05	4.1
TR19_NPPLTI_RI	SCONTIG63866_41	T	C	0.34	-14.64	7.5E-05	4.1
TR19_DFLRI_RI	SNODE_84004_43	T	G	0.23	1.14	7.7E-05	4.1
TRS18_HDS2M	SNODE_7070_46	T	G	0.09	0.91	7.9E-05	4.1
TR19_GYPLTC	SNODE_35312_45	A	T	0.38	5.77	7.9E-05	4.1
TR19_NPPLTI	SCONTIG106679_64	C	T	0.07	4.70	8.1E-05	4.1
TR19_NPPLTM	SNODE_134625_31	C	T	0.44	-2.15	8.1E-05	4.1
TR19_NSPI_RI	SNODE_2075_83	C	T	0.18	15.10	8.3E-05	4.1
TR19_DMATI	SNODE_3763_57	C	T	0.21	-0.81	8.4E-05	4.1
MR15_PLTHTM	SNODE_53396_78	G	T	0.23	7.29	8.4E-05	4.1
MR15_GYPLTC	SNODE_139623_44	A	T	0.36	12.78	8.5E-05	4.1
TR19_GCCM_RI	SCONTIG50118_65	G	A	0.08	-29.38	8.5E-05	4.1
TR16_DMATM_RI	SNODE_6679_53	T	A	0.48	0.71	8.9E-05	4.1
TR16_NPPLTTI_RI	***SNODE_239220_75***	G	C	0.41	30.20	8.9E-05	4.1
TR19_GYPLTM	SNODE_12261_51	C	T	0.06	-9.39	8.9E-05	4.0
TR19_GCCI_RI	SNODE_10934_67	A	G	0.35	-18.74	9.0E-05	4.0
MR15_PLTHTI_RI	SNODE_106637_40	C	T	0.07	73.72	9.1E-05	4.0
TR16_DFLRC	***SNODE_27970_52***	C	G	0.16	2.75	9.3E-05	4.0
TR19_GYPLTM_RI	SCONTIG7685_19	A	G	0.06	23.11	9.4E-05	4.0
TR16_DFLRI	SNODE_162178_22	A	G	0.07	8.88	9.5E-05	4.0
MR15_GYPLTM	SCONTIG61848_71	G	A	0.07	10.97	9.7E-05	4.0
TR19_DFLRM_RI	***SNODE_7114_58***	C	T	0.12	1.39	9.7E-05	4.0
TR16_DMATC	SNODE_12561_61	A	C	0.08	2.38	9.8E-05	4.0

Supplementary Table S2. Gene annotation information of some of the highly significant NPSs-traits associations.

SNP	Gene	Associated traits
CONTIG127798	acidic endochitinase	DFLR_RI; GCC_RI
NODE 14298 length 69 cov 191.202896	LRR receptor-like serine/threonine-protein kinase RCH1	PLHT_RI
NODE 3696 length 73 cov 344.041107	Probable serine/threonine-protein kinase NAK	GYPLT
NODE 4187 length 70 cov 94.728569	malate dehydrogenase	DFLR_RI
NODE 559376 length 95 cov 1.252632	photosystem I core protein PsaA	NPPLT_RI
NODE 7114 length 70 cov 20.785715	MYB-related protein P-like	DFLR

Supplementary Table S3**.** Details of the traits recorded at each experiment; traits are coded as the environment, followed by the trait, the treatment and "RI" if the number describe a reduction index.

**Environment**	**Trait**																	
	**DFLR**	**DFLR_RI**	**DMAT**	**DMAT_RI**	**NPPLT**	**NPPLT_RI**	**PLHT**	**PLHT_RI**	**NSPLT**	**NSPLT_RI**	**GYPLT**	**GYPLT_RI**	**NBrPLT**	**NBrPLT_RI**	**GCC**	**GCC_RI**	**HDS1**	**HDS2**
**Terbol 2016 Imazethapyr**	TR16DFLRI	TR16DFLRI_RI	TR16DMATI	TR16DMATI_RI	TR16NPPLTI	TR16NPPLTI_RI	TR16PTHTI	TR16PTHTI_RI	TR16NSPLTI	TR16NSPLTI_RI	TR16GYPLTI	TR16GYPLTI_RI	NA	NA	NA	NA	TR16HDS1I	TR16HDS2I
**Terbol 2016 Metribuzin**	TR16DFLRM	TR16DFLRM_RI	TR16DMATM	TR16DMATM_RI	TR16NPPLTM	TR16NPPLTM_RI	TR16PTHTM	TR16PTHTM_RI	TR16NSPLTM	TR16NSPLTM_RI	TR16GYPLTM	TR16GYPLTM_RI	NA	NA	NA	NA	TR16HDS1M	TR16HDS2M
**Terbol 2016 Control**	TR16DFLRC	NA	TR16DMATC	NA	TR16NPPLTC	NA	TR16PTHTC	NA	TR16NSPLTC	NA	TR16GYPLTC	NA	NA	NA	NA	NA	NA	NA
**Marchouch 2015 Imazethapyr**	NA	NA	NA	NA	NA	NA	RMR15PLTHTI	RMR15PLTHTI_RI	NA	NA	MR15GYPLTI	NA	NA	NA	NA	NA	NA	NA

Marchouch 2015 Metribuzin	Marchouch 2015 Control	Terbol 2019 Imazethapyr	Terbol 2019 Metribuzin	Terbol 2019 Control	Terbol Summer 2018 Metribuzin	Terbol Summer 2018 Control
NA	MR15DFLRC	TR19DFLRI	TR19DFLRM	TR19DFLRC	NA	NA
NA	NA	TR19DFLRI_RI	TR19DFLRM_RI	NA	NA	NA
NA	MR15DMATC	TR19DMATI	TR19DMATM	TR19DMATC	NA	NA
NA	NA	TR19DMATI_RI	TR19DMATM_RI	NA	NA	NA
NA	NA	TR19NPPLTI	TR19NPPLTM	TR19NPPLTC	NA	NA
NA	NA	TR19NPPLTI_RI	TR19NPPLTM_RI	NA	NA	NA
RMR15PLTHTM	TR19PLHTI	TR19PLHTM	TR19PLHTC	NA	TRS18PLHT1M	TRS18PLHT1C
RMR15PLTHTM_RI	NA	TR19PLHTM_RI	TR19PLHTC_RI	NA	TRS18PLHT1M_RI	NA
NA	NA	TR19NSPLTI	TR19NSPLTM	TR19NSPLTC	NA	NA
NA	NA	TR19NSPLTI_RI	TR19NSPLTM_RI	NA	NA	NA
MR15GYPLTM	MR15GYPLTC	NA	NA	NA	NA	NA
NA	NA	NA	NA	NA	NA	NA
NA	NA	TR19NBrPLTI	TR19NBrPLTM	TR19NBrPLTC	NA	NA
NA	NA	TR19NBrPLTI_RI	TR19NBrPLTM_RI	NA	NA	NA
NA	NA	TR19GCCI	TR19GCCM	TR19GCCC	TRS18GCCM	TRS18GCCC
NA	NA	TR19GCCI_RI	TR19GCCM_RI	NA	TRS18GCCM_RI	NA
NA	NA	NA	NA	NA	TRS18HDS1M	NA
MR15HDS2M	MR15HDS2I	TR19HDS2I	TR19HDS2M	NA	TRS18HDS2M	NA

DFLR days to flowering, DMAT days to maturity, NPPLT number of pods per plant, PLHT plant height, NSPLT number of seeds per plant, GYPLT grain yield per plant, NBrPLT number of branches per plant, GCC green canopy cover, HDS1 herbicide damage HDS2 recorded at the flowering stage, HDS2 herbicide damage HDS2 recorded at the pod development stage, RI Reduction index, NA not applicable.

Supplementary Table S4. Description of the damages observed in the treated plants for each herbicide damage score (HDS)

Herbicide damage score (HDS)	Description
**1**	No damage observed
	Normal phytosanitary status
	Normal and very good vegetative growth
**2**	Very light damage observed
	Very few leaf burnings
	Very good phytosanitary status
**3**	A clear moderate damage observed
	Stunting in growth with high yellowing
	Necrosis on leaves
**4**	A high damage was observed and death of <50% of plants
	Severe yellowing, leaf and stem burning with high deformations
	Very weak vegetative growth and stunted plants
**5**	Severe damage and death of >50% of plants
	High deformations and burnings
	High reduction of plant's biomass
	Overall yellowing was detected
